# Genetic Background, Maternal Age, and Interaction Effects Mediate Rates of Crossing Over in *Drosophila melanogaster* Females

**DOI:** 10.1534/g3.116.027631

**Published:** 2016-03-17

**Authors:** Chad M. Hunter, Matthew C. Robinson, David L. Aylor, Nadia D. Singh

**Affiliations:** * Program in Genetics, Department of Biological Sciences, North Carolina State University, Raleigh, North Carolina 27695; † W. M. Keck Center for Behavioral Biology, North Carolina State University, Raleigh, North Carolina 27695; ‡ Bioinformatics Research Center, North Carolina State University, Raleigh, North Carolina 27695; § Center for Human Health and the Environment, North Carolina State University, Raleigh, North Carolina 27695

**Keywords:** meiosis, recombination, aging

## Abstract

Meiotic recombination is a genetic process that is critical for proper chromosome segregation in many organisms. Despite being fundamental for organismal fitness, rates of crossing over vary greatly between taxa. Both genetic and environmental factors contribute to phenotypic variation in crossover frequency, as do genotype–environment interactions. Here, we test the hypothesis that maternal age influences rates of crossing over in a genotypic-specific manner. Using classical genetic techniques, we estimated rates of crossing over for individual *Drosophila melanogaster* females from five strains over their lifetime from a single mating event. We find that both age and genetic background significantly contribute to observed variation in recombination frequency, as do genotype–age interactions. We further find differences in the effect of age on recombination frequency in the two genomic regions surveyed. Our results highlight the complexity of recombination rate variation and reveal a new role of genotype by maternal age interactions in mediating recombination rate.

Meiotic recombination is a critically important biological process, as chromosomal crossovers are required for proper chromosome segregation in many organisms (Roeder 1997). Defects in meiotic recombination can have detrimental consequences, including increasing the probability of nondisjunction (Koehler *et al.* 1996; Hassold and Hunt 2001). The exchange of genetic material associated with crossing over can have important evolutionary consequences by combining or separating beneficial or deleterious alleles. Given the central importance of recombination for organismal fitness, one might hypothesize that this process would be highly regulated, with little to no variation present. However, a wealth of evidence in a variety of taxa points to the contrary. Variation in rates of recombination have been identified in yeast (Mancera *et al.* 2008), worms (Barnes *et al.* 1995; Rockman and Kruglyak 2009), fruit flies (Brooks and Marks 1986; Singh *et al.* 2009, 2013; Comeron *et al.* 2012), honey bees (Ross *et al.* 2015), maize (Bauer *et al.* 2013), chickens (Rahn and Solari 1986), mice (Dumont *et al.* 2009), chimpanzees (Ptak *et al.* 2005; Winckler *et al.* 2005), and humans (Kong *et al.* 2002; Crawford *et al.* 2004; Myers *et al.* 2005).

Though at least some of this variation is due to differences among genotypes, it has long been known that recombination rates are phenotypically plastic. That is, a given genotype has the capability to exhibit different phenotypes in response to different environmental conditions. For example, various types of stress have been associated with plastic increases in recombination rate, such as mating (Priest *et al.* 2007), nutrition (Neel 1941), parasitism (Singh *et al.* 2015), social stress (Belyaev and Borodin 1982), and temperature (Plough 1917, 1921; Stern 1926; Smith 1936; Grushko *et al.* 1991).

The effect of age on recombination rate has been investigated in some detail. This is likely because aging is a ubiquitous process, and one with often detrimental consequences. Indeed, for many organisms, advancing age is accompanied by a decrease in overall fitness (Williams 1957; Partridge and Barton 1993) and also a decrease in overall reproductive output (Stearns 1992). Many studies have examined how recombination changes with advancing maternal age in *Drosophila* (Bridges 1915, 1927, 1929; Plough 1917, 1921; Stern 1926; Bergner 1928; Neel 1941; Hayman and Parsons 1960; Redfield 1966; Lake and Cederberg 1984; Parsons 1988; Chadov *et al.* 2000; Priest *et al.* 2007; Tedman-Aucoin and Agrawal 2011; Stevison 2012; Manzano-Winkler *et al.* 2013; Hunter and Singh 2014). This topic has been investigated in other species as well, such as worms (Rose and Baillie 1979), tomatoes (Griffing and Langridge 1963), mice and hamsters (Henderson and Edwards 1968; Sugawara and Mikamo 1983), and humans (Kong *et al.* 2004; Coop *et al.* 2008; Hussin *et al.* 2011; Bleazard *et al.* 2013; Rowsey *et al.* 2014; Campbell *et al.* 2015; Martin *et al.* 2015).

In spite of the depth of research on this topic, a clear picture of how maternal age affects rates of recombination has yet to emerge. In humans, for instance, while some studies show fewer crossovers over time (*i.e.*, Kong *et al.* 2004; Hussin *et al.* 2011), others show more crossovers over time (*i.e.*, Tanzi *et al.* 1992; Bleazard *et al.* 2013; Martin *et al.* 2015). The *Drosophila* literature shows similar discrepancies, with some studies showing clear increases in crossover frequency with increasing maternal age (*i.e.*, Bridges 1915; Stern 1926; Bergner 1928; Lake and Cederberg 1984; Priest *et al.*, 2007; Hunter and Singh 2014), others showing decreases (*i.e.*, Bridges 1915; Hayman and Parsons 1960; Chadov *et al.*, 2000), some revealing nonlinear effects (*i.e.*, Plough, 1917, 1921; Bridges 1927; Neel 1941; Redfield 1966; Tedman-Aucoin and Agrawal 2011), and others yet finding no significant changes in recombination rates (*i.e*., Bridges 1915; Plough 1921; Stevison 2012; Manzano-Winkler *et al.*, 2013).

It has proven difficult to compare these studies for a variety of reasons, even within a single system such as *Drosophila*. First, many different strains have been employed in the above experiments, and it is not yet clear whether the effects of maternal age on recombination frequency are dependent on genetic background. Other factors, such as repeated mating, which may affect rates of crossing over in *Drosophila* (Priest *et al.* 2007), have not been controlled for in all studies, further complicating the interpretation of previous data. Experimental design differs among studies as well, with some studies assaying recombination from single females while others assay recombination from a pool of females; this too may contribute to the observed differences in the effects of maternal age on recombination among studies. Finally, different regions of the genome have been surveyed, and it is possible that the effect of maternal age on recombination rate is not uniform across the genome.

The goal of this study is to test the hypothesis that the effects of maternal age on recombination rate are genotype and/or locus-specific. Demonstrating genotype-by-age interaction effects or genomic heterogeneity in the magnitude/direction of age-associated changes in recombination rate is a critical first step in quantifying the extent of such effects and determining their genetic basis. To test for genotype–age interaction and locus-specific effects, we used multiple wild-type lines of *Drosophila melanogaster* and measured recombination rates of individual females for a period of 3 wk after a single mating event. This study estimated crossover rates in two different genomic locations. We find an increase of recombination rates with increasing maternal age on the X chromosome, though no significant age-dependency in recombination frequency on chromosome 3R. Our study confirms genotype-specific variation in recombination rate, and indicates that the effects of maternal age are indeed genotype-dependent. We also find a significant locus by maternal age effect, which suggests that age-related changes in recombination rate are likely to be variable across the genome. Our work establishes that it is important to control for genetic background effects when examining the effects of environmental factors on rates of crossing over. We predict that genotype–environment interaction effects on crossover rates are pervasive in other species as well.

## Materials and Methods

### Fly lines

Five inbred wild-type strains of *Drosophila* were used in this study from the *D. melanogaster* Genetic Reference Panel (DGRP) (Mackay *et al.* 2012; Huang *et al.* 2014). The five lines were RAL_21, RAL_59, RAL_73, RAL_75, and RAL_136. Four of the lines are free of chromosomal inversions and have the standard karyotype, while one (RAL_136) is heterozygous for both the Mourad inversion on 3L and the Kodani inversion on 3R (Huang *et al.* 2014). It should be noted that because of these inversions, RAL_136 was not used for estimating rates of recombination using markers on 3R. These lines were previously used in a study by the authors and were shown to be significantly genetically variable for crossover rates (Hunter and Singh 2014).

To measure rates of recombination, we employed a classical genetic crossing scheme using recessive visible markers. The markers used to measure recombination on the X chromosome were *yellow* (*y*^1^) and *vermilion* (*v*^1^) (Bloomington Drosophila Stock Center #1509), which are 33 cM apart (Morgan and Bridges 1916), integrated into a wild-type isogenic Samarkand genetic background (Lyman *et al.* 1996); this line abbreviated hereafter as ‘*y v.*’ The markers on the 3R chromosome were *ebony* (*e*^4^) and *rough* (*ro*^1^) (Bloomington Drosophila Stock Center #496), which are 20.4 cM apart (Bridges and Morgan 1923); this line is abbreviated hereafter as ‘*e ro*.’ These markers were selected due to their genetic distance, ease of scoring, and lack of viability defects. To assay rates of nondisjunction, we used a multiply marked fly strain. The full genotype of this strain is *y cv v f* / *T(1:Y)B^S^* (Kohl *et al.* 2012).

### Experimental crosses

All crosses were executed at 25° with a 12 hr:12 hr light:dark cycle on standard cornmeal-molasses media. To score crossover frequency, we used a two-step crossing scheme (Supplemental Material, Figure S1). For the first cross, 20 virgin DGRP females were mated to 20 doubly-marked males for 5 d in 8 ounce (oz.) bottles (doubly-marked males are denoted by *m*_1_ *m*_2_ for simplicity and refer to either *y v* males or *e ro* males). After 5 d, parental flies were removed. Virgin F_1_ females (+ +/ *m*_1_ *m*_2_) were collected within a 2 hr period between 8 am and 10 am on the same day for all lines and held virgin for 24 hr in groups of 20. Twenty virgin females were mass-mated with 20 males in 8 oz. bottles for a period of 24 hr (for flies mated to *y v* males) or for 48 hr (for flies mated to *e ro* males). Flies used for the *e ro* cross produced very few gravid females in a first trial of a 24 hr window, necessitating the longer mating window. Due to the apparent effect of repeated mating on rates of recombination (Priest *et al.* 2007), we limited females to mating attempts only in the short window of 24–48 hr. This short window allows for roughly one mating event since females become unresponsive to remating for roughly 1 d after copulation (Manning 1962, 1967; Gromko *et al.* 1984). Drosophila females are able to store sperm for periods greater than 2 wk (Kaufman and Demerec 1942; Lefevre and Jonsson 1962) so all progeny collected are the result of mating within that original 24–48 hr window. After mating, individual females were placed into vials and transferred every 2 d at the same time of day for 22 d. We conducted this experiment twice; once for the *y v* marker pair and once for the *e ro* marker pair. For *y v*, 150 replicate females were used for each line. For *e ro*, 175 replicate females were used for each line. The resulting progeny from each vial were scored for both sex and the presence of morphological markers. Recombinant progeny were identified by the presence of only one visible marker (recombinant genotypes are *m*_1_ + or + *m*_2_). Table S1 and Table S2 contain progeny counts from individual females for each phenotype class from each day in each interval. Table S3 summarizes these data across lines for a given time point and interval.

To assay rates of nondisjunction, we used a simple crossing scheme (Figure S2). All crosses were executed at 25° with a 12 hr:12 hr light:dark cycle on standard media using virgin females aged roughly 24 hr. For the cross, 10 or 20 (depending on how many virgins eclosed on a given day) virgin females from each line were crossed to the same number of *y cv v f* / *T(1:Y)B^S^* males in 8 oz. bottles. Males and females were transferred to fresh bottles every 5 d for a total of 25 d. All progeny were collected and scored for both sex and presence/absence of Bar (*B^S^*) eyes. Females displaying Bar eyes or males displaying wild-type eyes indicated a nondisjunction event. The total number of nondisjunction progeny observed was multiplied by two to account for triplo-X and nullo-X progeny, which are lethal (and thus not observable). Table S4 summarizes these data across lines for a given time point and interval.

### Statistics

All statistics were conducted using JMPPro v11.0.0 and/or R v3.2.0 unless otherwise noted. We used a repeated measures ANOVA (Winer 1971) on arcsine square root transformed data and tested for the effects of maternal age, genetic background, and the interaction between these factors. The full model is as follows:

$$\begin{array}{c} R_{\text{ij}}=\mu +G_{\text{i}}+A_{j}+I_{ij}^{\left(G\times A\right)}+\varepsilon_{k}+\mathrm{\rho ,}\\ \text{for}\;y\;v,i=1\ldots 5;j=1\ldots 6;\text{and}\;k=1\ldots 307\\ \text{and}\;\;\text{for}\;e\;ro,i=1\ldots 2;j=1\ldots 3;\text{and}\;k=1\ldots 54 \end{array}$$

where *R* represents (transformed) crossover frequency, μ represents the mean of regression, ε represents the individual error, and ρ represents the residual error. *G* represents female genetic background, *A* represents maternal age, and *I^(G×A)^* represents the interaction of the two. Each of these terms was modeled as a fixed effect. For the repeated measures ANOVA, we restricted our analysis to days 1–12 for the interval on the X chromosome, because the number of progeny produced markedly decreased after day 12 (over a threefold decrease comparing the average of days 1–12 to the average of days 14–22; Table S3). Similarly, we limited our analysis to days 1–10 for the interval on 3R for the same reason (Table S3).

Additionally, we used a generalized linear model with a binomial distribution and logit link function on the proportion of progeny that are recombinant. We treated each offspring as a realization of a binomial process (either recombinant or nonrecombinant), summarized the data for a given vial by the number of recombinants and the number of trials (total number of progeny per vial), and tested for an effect of age, genetic background, and the interaction of the two. The full model was as follows:

$$\begin{array}{c} Y_{\text{ij}}=\mu +G_{\text{i}}+A_{\text{j}}+I_{ij}^{\left(G\times A\right)}+\varepsilon_{k},\\ \text{for}\;y\;v:i=1\ldots 5,j=1\ldots 10,\text{and}\;k=1\ldots 2648\\ \text{and}\;\;\text{for}\;e\;ro:i=1\ldots 4,j=1\ldots 3,\text{and}\;k=1\ldots 625 \end{array}$$

where *Y* represents the proportion of progeny that is recombinant, μ represents the mean of regression, and ε represents the error. Once again, *G* represents female genetic background, *A* represents maternal age, and *I^(G×A)^* represents the interaction of the two, all modeled as fixed effects.

To test for locus effects, we used the same generalized linear model as detailed above, (once again, with a binomial distribution and logit link function) to test for an effect of age, genetic background, and also locus, as well as all possible interactions. The full model is as follows:

$$\begin{array}{c} Y_{\text{ij}}=\mu +G_{i}+A_{j}+L_{k}+I_{ij}^{\left(G\times A\right)}+I_{ik}^{\left(G\times L\right)}+I_{jk}^{\left(A\times L\right)}+I_{ijk}^{\left(G\times A\times L\right)}+\varepsilon_{k},\\ \text{where}\;i=1\ldots 4;j=1\ldots 3;k=1\ldots 2;\text{and}\;k=1\ldots 1927 \end{array}$$

where *Y* represents the proportion of recombination progeny and μ represents the mean of regression. *G* represents female genetic background, *A* represents maternal age, and *L* represent locus assayed (either *y v* or *e ro*), all modeled as a fixed effects, along with all interaction terms. Data points included three maternal ages (days 2, 4, and 6–10) for both loci.

We used a generalized linear model with a binomial distribution and logit link function to test for an effect of age, genetic background, as well as the interaction of the two on the proportion of progeny that are aneuploid. We treated each offspring as a realization of a binomial process (euploid *vs.* aneuploid), and summarized the data for a given bottle by the number of aneuploid progeny (multiplied by two to account for triplo-X and nullo-X progeny which are lethal) and the number of trials (total number of progeny per bottle plus unobservable lethal progeny). The full model was as follows:

$$\begin{array}{c} Y_{\text{ij}}=\mu +G_{\text{i}}+A_{\text{j}}+I_{ij}^{\left(G\times A\right)}+\varepsilon_{k},\\ i=1\ldots 5,j=1\ldots 5,\text{and}\;k=1\ldots 150 \end{array}$$

where *Y* represents the proportion of aneuploid progeny, μ represents the mean of regression, and ε represents the error. *G* represents female genetic background, modeled as a fixed effect, and *A* represents maternal age, also modeled as a fixed effect, along with the interaction of the two (*I^(G×A)^*).

### Data availability

The authors state that all data necessary for confirming the conclusions presented in the article are represented fully within the article.

## Results and Discussion

### Robustness of crossover frequency estimation

In total, we scored 105,378 progeny for both intervals combined (78,292 for the *y v* interval and 27,086 for the *e ro* interval). We performed G-tests for goodness of fit (Sokal and Rohlf 1994) on our combined data to validate that the correct proportions of females *vs.* males, wild-type *vs. m*_1_ *m*_2_, and *m*_1_ + *vs.* + *m*_2_ were being recovered. It is expected that each of these pairs will be recovered in a 1:1 ratio due to Mendelian segregation. Comparing females *vs.* males for the *y v* interval, only 1 out of 613 replicates showed a significant deviation from the 1:1 ratio (Bonferroni-corrected *P* = 0.05, *G*-test) while for the *e ro* interval, 0 out of 467 replicates showed a significant deviation from the 1:1 ratio (Bonferroni-corrected *P* > 0.05, all comparisons, *G*-test). Comparing wild-type *vs. m*_1_ *m*_2_ (progeny with both markers) in the *y v* interval, 6 out of 613 replicates showed a significant deviation from the expected 1:1 ratio (Bonferroni-corrected *P* < 0.05, *G*-test), while for the *e ro* interval, none of the replicates showed a significant deviation from the 1:1 ratio (Bonferroni-corrected *P* > 0.05, all comparisons, *G*-test). Comparing the ratio of recombinant progeny (*m*_1_ + *vs.* + *m*_2_), none of the replicates showed a significant deviation from the expected 1:1 ratio for the either the *y v* or *e ro* interval (Bonferroni-corrected *P* > 0.05, all comparisons, *G*-test). These results indicate that there is no viability defect associated with any of the mutations used in the current study and gives us confidence that our estimates of crossover are robust.

### Interaction of genetic background and maternal age

The primary motivation for this study was to determine how crossover frequency varies in relation to genetic backgrounds, advancing maternal age, and the interaction of the two. Although work has shown that meiotic nondisjunction increases with maternal age in *Drosophila* (using oocytes aged ∼4 d; Jeffreys *et al.* 2003; Subramanian and Bickel 2008, 2009; Weng *et al.* 2014), the nature of the relationship between recombination rate and maternal age is less clear. As described before, increases, decreases, nonlinear, and no changes in rates of recombination with increasing maternal age have all been observed previously.

We used a repeated measures ANOVA to test for significant effects of genetic background, maternal age, and the interaction of age and genotype on recombination frequency data from individual females. Repeated measures ANOVA are uniquely well-suited to the longitudinal structure of our data—recombination rate measurements from the same individuals at multiple timepoints. Although our residuals after model-fitting show significant deviations from normality (*P* = 0.01, Kolmogorov–Smirnov test), ANOVAs are robust even when assumptions are of the model are violated (Glass *et al.* 1972; Schmider *et al.* 2010). Thus, a repeated measures ANOVA is an appropriate framework in which to analyze these data, given our focus on the role of age on recombination rate. However, we couple this approach with an additional type of analysis (see below) to ensure that our findings are robust.

**Figure 1:**
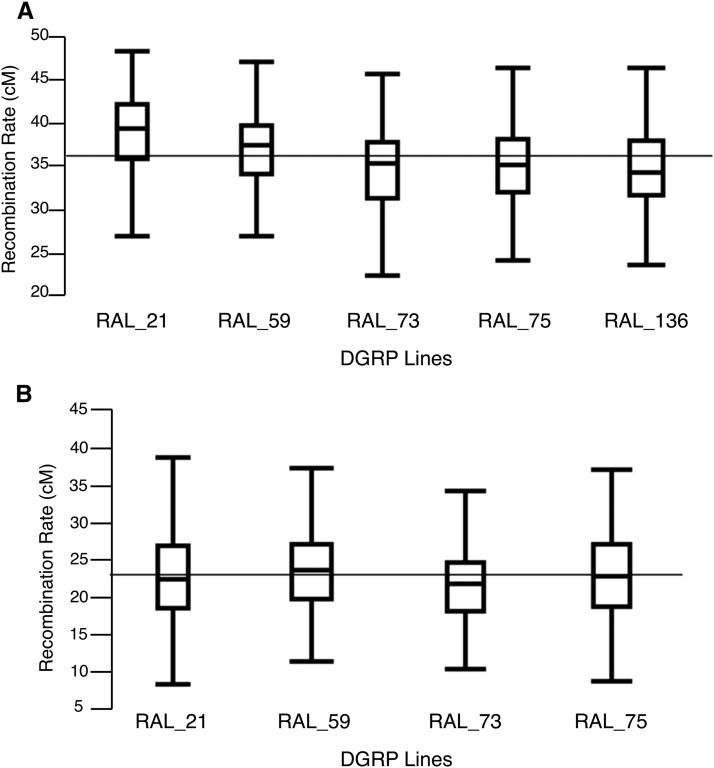
Crossover frequency summed across an individual female’s lifetime for the (A) *y v* interval or (B) *e ro* interval. Boxplots show first to third quartiles with median denoted by line inside the box with whiskers extending to the smallest and largest nonoutliers, while the gray line indicates the grand mean.

For the *y v* region data (up to 12 d; see *Materials and Methods*), the repeated measures ANOVA reveals that genetic background (*F*_4,302_ = 10.86; *P* < 0.001; Table 1) significantly contributes to the recombination rate observed in our study. This is consistent with previous work in *Drosophila*, which has also highlighted a role of genetic variation in mediating crossover frequency both within the DGRP lines specifically (Comeron *et al.* 2012; Hunter and Singh 2014; Hunter *et al.* 2016) as well as in *Drosophila* in general (Chinnici 1971a,b; Brooks and Marks 1986; Comeron *et al.* 2012). Moreover, the magnitude of variation in recombination rate that we observe across lines (∼1.6-fold in the current study; Figure 1) is consistent with the magnitude of interstrain variability in Drosophila (∼1.3-fold; Brooks and Marks 1986; Hunter and Singh 2014; Hunter *et al.* 2016) A role for genetic background in recombination rate variation is seen in other species as well, including mice (*e.g.*, Dumont *et al.* 2009; Dumont and Payseur 2011) and humans (*e.g.*, McVean *et al.* 2004; Fearnhead and Smith 2005; Graffelman *et al.* 2007; Kong *et al.* 2010).

**Table 1:** Results from repeated measures ANOVA to test for significant effects of genetic background (line), age, and their interaction on crossover frequency in the two intervals assayed

Chromosome	Source	df	SS	MS	*F*-Value	Prob > *F*
X	Line	4	1.34	0.34	10.25	< 0.001
	Residuals	305	9.96	0.033		
	Maternal age	1	1.32	1.32	54.19	< 0.001
	Line × maternal age	4	0.66	0.17	6.78	< 0.001
	Residuals	1855	45.19	0.024		
3R	Line	2	0.0011	0.00059	0.033	0.97
	Residuals	15	0.27	0.018		
	Maternal age	1	0.046	0.046	2.93	0.097
	Line × maternal age	2	0.069	0.0035	0.22	0.80
	Residuals	33	0.52	0.016		

df, degrees of freedom; SS, sum of squares; MS, mean square.

**Figure 2:**
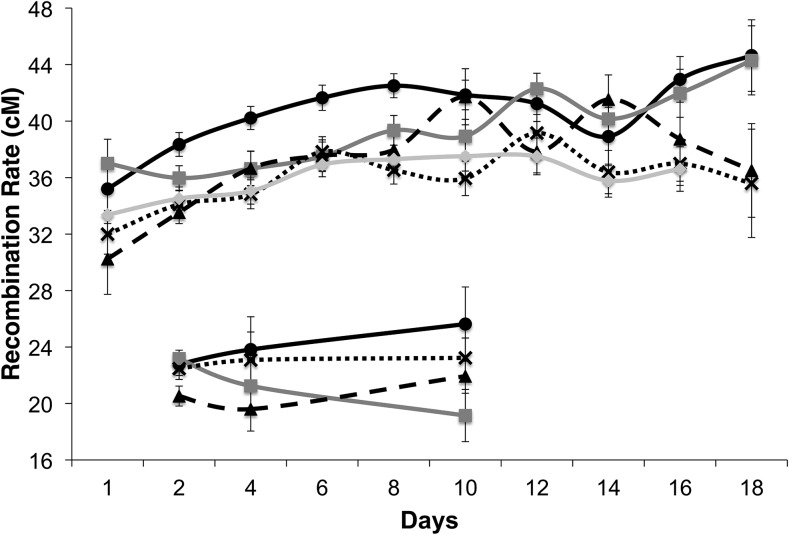
Average crossover frequency separated by day for RAL_21 (black line, ● data points), RAL_59 (dark gray line, ▪ data points), RAL_73 (long-dashed black line, ▴ data points), RAL_75 (short-dashed black line, X data points), and RAL_136 (light gray line, ♦ data points). Upper lines represent crossover frequency in the *y v* interval while lower lines represent crossover frequency in the *e ro* interval. Error bars denote standard error.

Our results indicate that maternal age also contributes to variation in recombination rate observed in the current study (F_1,1837_ = 56.09; *P* < 0.001). Our data further indicate that rates of crossing over increase with maternal age within the *y v* genomic region (Figure 2), although these increases appear to not be strictly linear. The increase in recombination frequency with increasing maternal age is consistent with several previous studies in *Drosophila* (Bridges 1915; Stern 1926; Bergner 1928; Lake and Cederberg 1984; Priest *et al.* 2007; Hunter and Singh 2014) and other species such as humans (Kong *et al.* 2004; Coop *et al.* 2008; Martin *et al.* 2015).

In humans, increased recombination with increasing age is associated with a reduced incidence of aneuploidy (Ottolini *et al*. 2015). Estimating levels of nondisjunction of these same five DGRP lines over a 25 d period (Table S4), we observe no significant effect of age (*P* = 1), yet we do observe a significant effect of genetic background and the interaction of genetic background and age (*P* < 0.001, both factors; Table S5). These results suggest that, like rates of recombination, different genetic backgrounds also vary in their amount of nondisjunction. Thus, it appears that although both *Drosophila* and humans can show increases in recombination with increasing maternal age, rates of aneuploidy are less dependent on age *per se* and more dependent on genetic background in *Drosophila*.

Central to our motivating hypothesis, the interaction of genetic background and maternal age also significantly contributes to phenotypic variation in recombination rate (*F*_4,1837_ = 6.45; *P* < 0.001; Table 1). This indicates that the effects of maternal age on recombination rate are genotype-dependent. While previous work showed that different strains of *D. melanogaster* containing different dominant deleterious mutations differed in the magnitude and extent of age-dependent changes in recombination (Tedman-Aucoin and Agrawal 2011), here we report that natural genetic variation can also drive changes in the effects of maternal age on recombination rate.

To assess the robustness of our findings, we tested for effects of maternal age, genetic background, and genotype–age interactions using a generalized linear model. While this statistical approach does not require that residuals are normally-distributed as the ANOVA framework does, it does not capture the repeated measurement structure of our data when partitioning variance. Analysis of the full data complement for the *y v* interval using a generalized linear model reveals significant effects of line and maternal age (*P* < 0.001 for both factors), and a marginally significant effect of genotype-by-age interaction on recombination rate variation (Table 2). The marginal significance revealed by this logistic regression, coupled with the high significance revealed by the repeated measures ANOVA, indicate that our results are largely robust to statistical approach and, moreover, are consistent with a statistically significant line by age interaction effect. As a further test of robustness, we repeated both the repeated measures ANOVA and the logistic regression after removing RAL_136 (which contains segregating inversions on arms 3L and 3R (see *Materials and Methods*)); these analyses produce the same results in both cases (Table S6), indicating that this line is not driving the effect.

**Table 2:** Results from generalized linear model to test for effects of genetic background (line), age, and their interaction on crossover frequency in the two intervals assayed

Chromosome	Source	df	*χ*^2^	Prob > *χ*^2^
X	Line	4	46.41	< 0.001
	Maternal age	9	126.10	< 0.001
	Line × maternal age	36	48.80	0.075
3R	Line	3	7.84	0.0495
	Maternal age	2	0.039	0.98
	Line × maternal age	6	4.22	0.65

df, degrees of freedom, *χ*^2^, chi-square value.

It bears mentioning that our surveyed window does not fully capture the potential effects of age on recombination. Indeed, *Drosophila* can have lifespans of ∼80 d and beyond (Grönke *et al.* 2010; Mockett *et al.* 2012; Ivanov *et al.* 2015). However, the average lifespan is ∼45–60 d under optimal conditions (see Ivanov *et al.*, 2015), and usually less under normal conditions (Ashburner *et al.* 2005). Additionally, the act of mating can significantly reduce the average lifespan of a female as compared to her nonmated counterpart (Fowler and Partridge 1989). The average (unmated) lifespan for the five lines used in this study is ∼56 d (Arya *et al.* 2010; Ivanov *et al.* 2015). Therefore, our measurements spanning 22 d encompass a large proportion of the adult lives of these flies. While it is possible that were we able to survey recombination rates over a longer period of time we would see more dramatic effects of age on recombination, that we observe a significant effect of maternal age on recombination rates in the *y v* region indicates that the effects of age, even within the first 22 d, are biologically significant.

### Locus effects

Previous research has indicated that rates of crossing vary along the genome, both on broad and fine scales (Lindsley *et al.* 1977; McVean *et al.* 2004; Cirulli *et al.* 2007; Paigen *et al.* 2008; Singh *et al.* 2009, 2013; Comeron *et al.* 2012). We hypothesized that changes in crossover frequency due to age might also be variable across the genome, and another goal of this work was to test the whether the effects of maternal age on recombination frequency are locus-dependent. By using markers on both the X and 3R chromosomes, we can compare the effect of maternal age and genetic background at two different genomic locations. For the recombination rate estimation on chromosome 3R, we limited our analysis to only the first 10 d, combining progeny from days 6–10. This maximized the useable data, as we recovered fewer progeny overall from this crossing scheme as compared with the crossing scheme used to survey recombination on the X chromosome. In addition, we did not include RAL_136 in this experiment due to the aforementioned segregating inversions.

A repeated measures ANOVA of the *e ro* region data suggests no factors are significant (Table 1). Using a generalized linear model (see *Materials and Methods*), we find that genetic background significantly contributes to the observed variation in recombination rate (*P* = 0.05), but neither maternal age (*P* = 0.98) nor the interaction term (*P* = 0.65) are significant. Once again, the lifetime measure of recombination (as calculated from all progeny from an individual female over her lifetime) varies ∼2.5-fold (Figure 1B), which is on the same scale as the *y v* region as well as previous work (Brooks and Marks 1986; Hunter and Singh 2014; Hunter *et al.* 2016). Given the sensitivity of these results to the method of analysis, it is difficult to interpret the results. However, it is worth noting that reducing the X chromosome dataset to the first 10 d only and combining days 6–10 confirms significant effects of genetic background (*P* < 0.001), maternal age (*P* < 0.001), and the interaction of the two (*P* = 0.02) on recombination frequency in this X chromosome interval using a repeated measures ANOVA, both with and without DGRP_136 (Table S7). This indicates that the lack of a detectable effect of maternal age on crossover frequency on 3R is not due to the sampling structure of the experiment. That we detect no consistent effect of age on recombination frequency in the third chromosome region surveyed is suggestive that crossover frequency at this locus is differentially sensitive to environmental variation.

To test explicitly for a locus effect, we used a generalized linear model with a binomial distribution and logit link function using data up to day 10 from both loci (see *Materials and Methods*) to test for significant effects of genetic background, maternal age, and locus, and their interactions. We observe a significant effect of genetic background, maternal age, locus, and maternal age × locus (*P* < 0.02 for all factors) and a marginally significant effect genetic background × locus of (*P* = 0.08) (Table 3). The significant effect of maternal age × locus suggests that the effects of age on recombination frequency are significantly variable across the genome.

**Table 3:** Results from generalized linear model to test for significant effects of genetic background (line), age, locus, and their interactions on crossover frequency using a combined model to test for locus and locus interaction effects

Source	df	*χ*^2^	Prob > *χ*^2^
Line	3	23.98	< 0.001
Locus	1	705.42	< 0.001
Maternal age	2	7.08	0.029
Line × locus	3	6.63	0.084
Locus × maternal age	2	7.69	0.021
Line × maternal age	6	4.38	0.63
Line × maternal age × locus	6	3.59	0.73

df, degrees of freedom; *χ*^2^, chi-square value.

Integrating our findings with previous work also points to genomic heterogeneity in the recombinational response to maternal age. For instance, data in humans are similarly suggestive of chromosome-level variability in the effect of maternal age on crossover frequency (Hussin *et al.* 2011). Moreover, Bridges (1915) found differences in the frequency of crossing over in two different broods from the same *D. melanogaster* females for markers on the third chromosome (*pink* and *kidney*), but no significant differences in crossover frequency in broods between markers on the X chromosome (*vermilion* and *fused*). Interestingly, our results show the opposite: significant increases in recombination on the X chromosome but no significant changes in recombination rate on chromosome 3. These data hint at the possibility that not only does the effect of maternal age on recombination vary as a function of genomic position, but that it may also vary depending on the genetic background of the strain surveyed.

We uncover neither a significant line by locus by age interaction effect nor a significant line by maternal age interaction effect on recombination frequency in the current study (Table 3). However, we are likely underpowered to do so. By increasing both the number of genomic intervals and the number of genetic backgrounds analyzed, one might be better able to detect these interaction effects, which appear to be weaker than the effects of factors such as genetic background and maternal age. Additionally, increasing the sample size by allowing repeated mating would increase the number of progeny produced by individual females, adding power to the analyses. Surveying additional females could also add power and could facilitate uncovering such interaction effects.

It should also be pointed out that the markers used in this study are both distal in location, so it is somewhat surprising that they show different trends. It is possible that the use of markers more proximal to the centromere or in other chromosomal locations could show different results, as distribution of recombination is not uniform along the length of chromosomes (Charlesworth and Campos 2014). Future studies will be aimed at analyzing how rates of recombination respond to advancing maternal age across the entirety of the genome, allowing for tests of differences between distal and proximal regions of chromosomes.

### Conclusions

Our results indicate that crossover frequency is mediated by genetic background and maternal age. The novel contribution of our work is the finding of natural genetic variation for age-dependent changes in recombination rate in *Drosophila*. Future work will be aimed at quantifying the magnitude of genotype–age interaction effects in natural populations. Moreover, the DGRP provides a community resource that could potentially be used to uncover the genetic basis of these interaction effects, another area of future work. Our data are also indicative of genomic variability in the effects of maternal age on recombination frequency, opening the possibility that environmental stressors may influence different parts of the genome in different ways. Future work will also be aimed testing for heterogeneity in the recombinational response to environmental stimuli at a genomic scale.

## Supplementary Material

Supplemental Material

Supplemental Material
