# Supplementary material for: Genetic Background, Maternal Age, and Interaction Effects Mediate Rates of Crossing Over in Drosophila melanogaster Females
Source: G3 (Bethesda). 2016 Mar 17;6(5):1409–16. doi: 10.1534/g3.116.027631 (PMC4856091; doi:10.1534/g3.116.027631)
Supplement: Supplemental Material [file supp_6_5_1409__index.html]

Genetic Background, Maternal Age, and Interaction Effects Mediate Rates of Crossing Over in Drosophila melanogaster Females — Supplemental Material 

# Genetic Background, Maternal Age, and Interaction Effects Mediate Rates of Crossing Over in *Drosophila melanogaster* Females

## Supplemental Material for Hunter *et al.*, 2016

**Files in this Data Supplement:**

- Figure S1 - Two-step crossing scheme used in order to generate crossover frequency in the (A) *e ro* and (B) *y v* region. (.tiff, 219 KB)
- Figure S2 - Crossing scheme to measure nondisjunction in *D. melanogaster*. (.tiff, 133 KB)
- Table S1 - Progeny counts for each phenotype (+ +, y1 v1, + v1, and y1 +) for each sex for the five lines (DGRP\_21, DGRP\_59, DGRP\_73, DGRP\_75, and DGRP\_136) assayed for recombination over 22-day period. (.xlsx, 426 KB)
- Table S2  - Progeny counts for each phenotype (+ +, *e*4*ro*1, + *ro*1, and *e*4 +) for each sex for the four lines (DGRP\_21, DGRP\_59, DGRP\_73, and DGRP\_75) assayed for recombination over 22-day period. (.xlsx, 361 KB)
- Table S3 - Number of total progeny scored per day for the interval on the X chromosome (*y*1*v*1) and chromosome 3R (*e*4*ro*1) intervals. (.xlsx, 40 KB)
- Table S4 - Progeny counts for each phenotype (wild-type or Bar eyed) for each sex for the five lines (DGRP\_21, DGRP\_59, DGRP\_73, and DGRP\_75, DGRP\_136) assayed for nondisjunction over 25-day period. (.xlsx, 49 KB)
- Table S5 - Results from generalized linear model to test for significant effects of genetic background (line), age, and their interaction on nondisjunction frequency over time. (.xlsx, 34 KB)
- Table S6 - Results from repeated measures ANOVA as well as generalized linear model to test for significant effects of genetic background (line), age, and their interaction on crossover frequency in the X interval with DGRP\_136 removed due to chromosomal inversions. (.xlsx, 40 KB)
- Table S7 - Results from repeated measures ANOVA to test for significant effects of genetic background (line), age, and their interaction on crossover frequency in the X collapsing days 6-10. (.xlsx, 41 KB)
