# Supplementary figures and images for: Genetic Background, Maternal Age, and Interaction Effects Mediate Rates of Crossing Over in Drosophila melanogaster Females
Source: G3 (Bethesda). 2016 Mar 17;6(5):1409–16. doi: 10.1534/g3.116.027631 (PMC4856091; doi:10.1534/g3.116.027631)

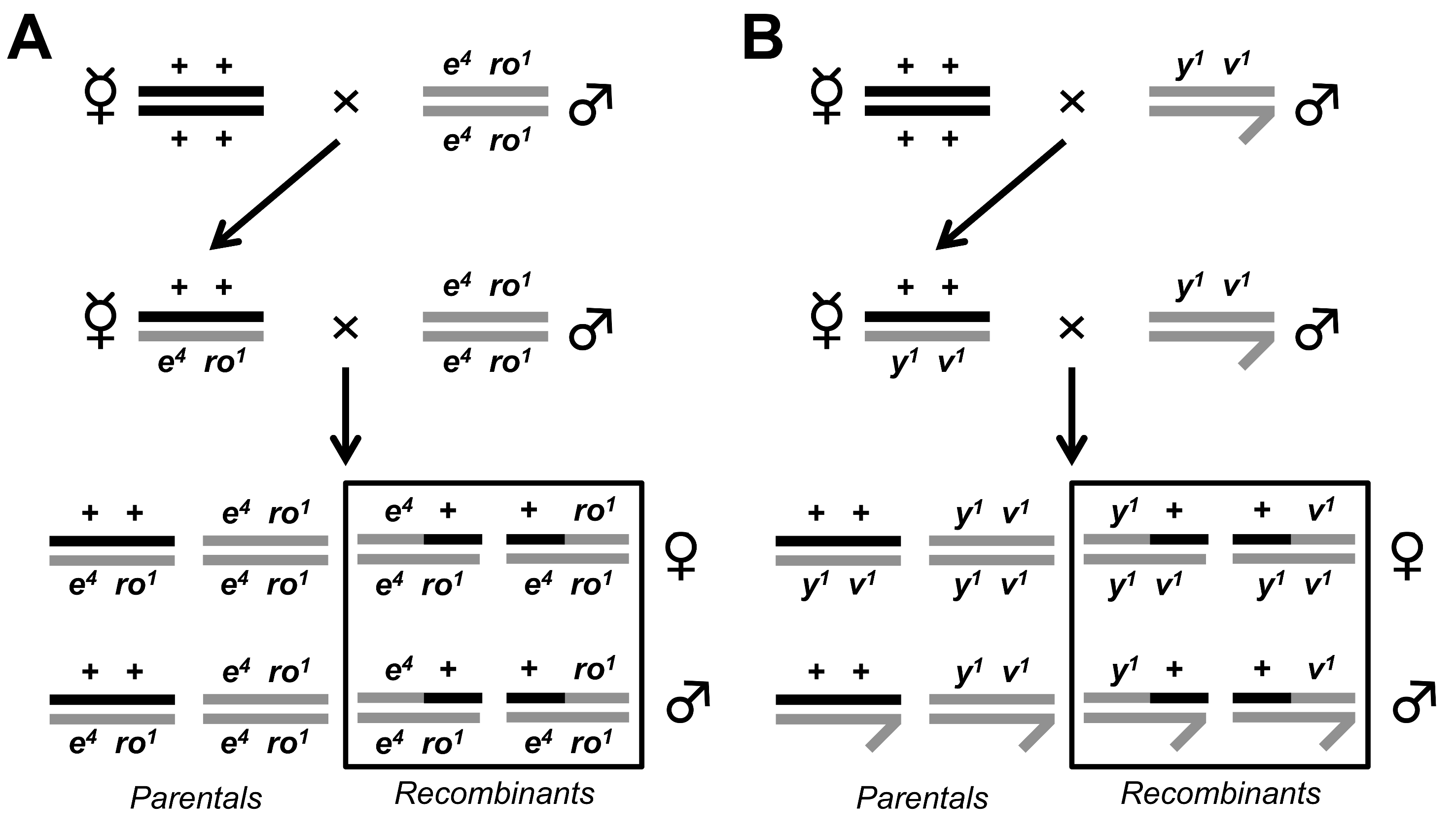

Supplement: Supplemental Material [file supp_g3.116.027631_FigureS1.tif]

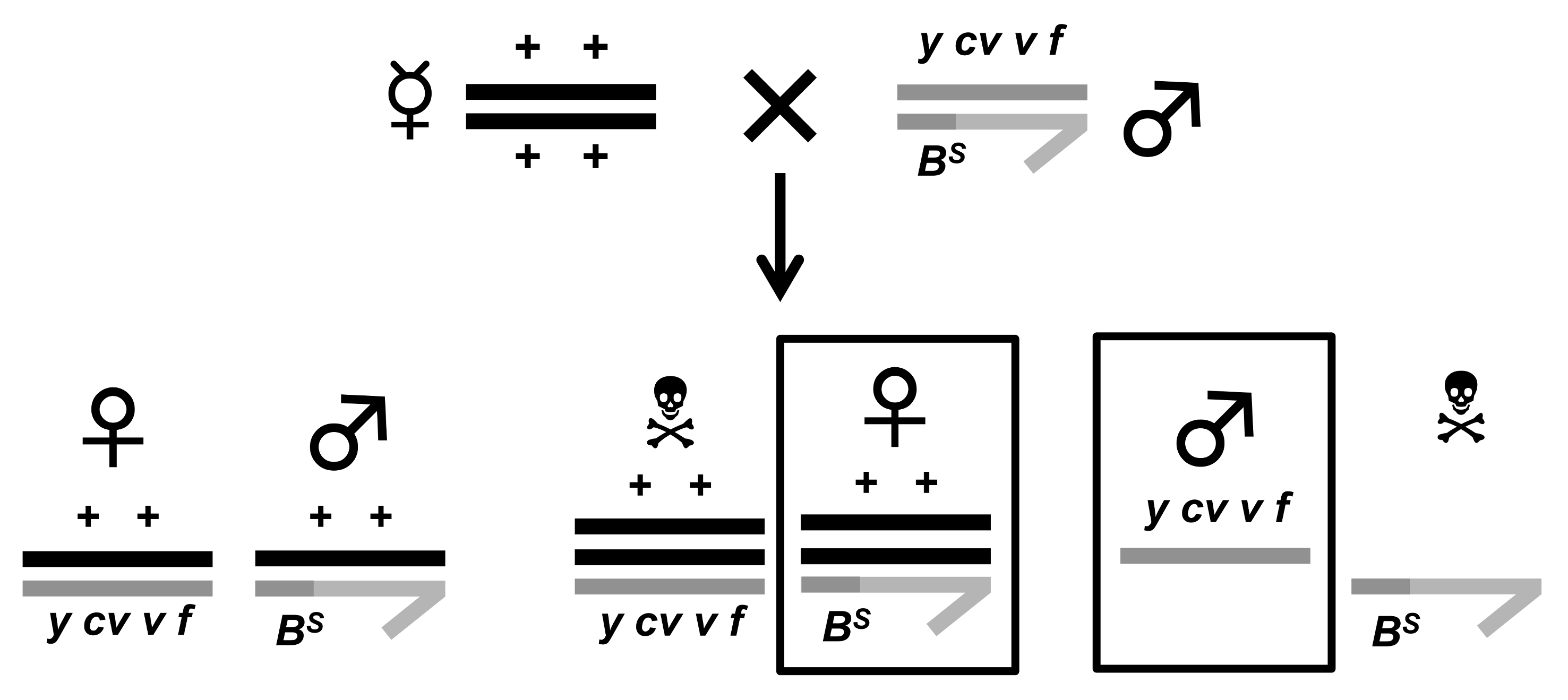

Supplement: Supplemental Material [file supp_g3.116.027631_FigureS2.tif]
